# Tetanus: Pathophysiology, Treatment, and the Possibility of Using Botulinum Toxin against Tetanus-Induced Rigidity and Spasms

**DOI:** 10.3390/toxins5010073

**Published:** 2013-01-08

**Authors:** Bjørnar Hassel

**Affiliations:** 1 Norwegian Defense Research Establishment, N-2027 Kjeller, Norway; E-Mail: bjornar.hassel@ffi.no; Tel.: +47-63-807-846; Fax: +47-63-807-509; 2 Department of Neurology, Oslo University Hospital-Rikshospitalet, 0027 Oslo, Norway

**Keywords:** tetanus, tetanospasmin, *Clostridium tetani*, botulinum toxin, trismus, lockjaw, dysphagia

## Abstract

Tetanus toxin, the product of *Clostridium tetani*, is the cause of tetanus symptoms. Tetanus toxin is taken up into terminals of lower motor neurons and transported axonally to the spinal cord and/or brainstem. Here the toxin moves trans-synaptically into inhibitory nerve terminals, where vesicular release of inhibitory neurotransmitters becomes blocked, leading to disinhibition of lower motor neurons. Muscle rigidity and spasms ensue, often manifesting as trismus/lockjaw, dysphagia, opistotonus, or rigidity and spasms of respiratory, laryngeal, and abdominal muscles, which may cause respiratory failure. Botulinum toxin, in contrast, largely remains in lower motor neuron terminals, inhibiting acetylcholine release and muscle activity. Therefore, botulinum toxin may reduce tetanus symptoms. Trismus may be treated with botulinum toxin injections into the masseter and temporalis muscles. This should probably be done early in the course of tetanus to reduce the risk of pulmonary aspiration, involuntary tongue biting, anorexia and dental caries. Other muscle groups are also amenable to botulinum toxin treatment. Six tetanus patients have been successfully treated with botulinum toxin A. This review discusses the use of botulinum toxin for tetanus in the context of the pathophysiology, symptomatology, and medical treatment of *Clostridium tetani* infection.

## 1. Introduction

The muscular rigidity and spasms of tetanus are caused by tetanus toxin (tetanospasmin), which is produced by *Clostridium tetani*, an anaerobic bacillus, whose spores survive in soil and cause infection by contaminating wounds [1]. The global incidence of tetanus has been estimated at approximately one million cases annually [1,2]. Mortality rates from tetanus vary greatly across the world, depending on access to healthcare, and approach 100% in the absence of medical treatment [3]. This review discusses the possibility of using botulinum toxin for tetanus-induced rigidity and spasms in the context of the pathophysiology, symptomatology, and medical treatment of *Clostridium tetani* infection.

## 2. Pathophysiology of Tetanus Toxin

By a mechanism similar to that of botulinum toxin, tetanus toxin is taken up into nerve terminals of lower motor neurons, the nerve cells that activate voluntary muscles [4,5,6]. Tetanus toxin is a zinc-dependent metalloproteinase that targets a protein (synaptobrevin/vesicle-associated membrane protein—VAMP) that is necessary for the release of neurotransmitter from nerve endings through fusion of synaptic vesicles with the neuronal plasma membrane [7]. The initial symptom of local tetanus infection may therefore be flaccid paralysis [8,9], caused by interference with vesicular release of acetylcholine at the neuromuscular junction, as occurs with botulinum toxin. However, unlike botulinum toxin, tetanus toxin undergoes extensive retrograde transport in the axons of lower motor neurons and thus reaches the spinal cord or brainstem [3,7]. Here, the toxin is transported across synapses and taken up by nerve endings of inhibitory GABAergic and/or glycinergic neurons that control the activity of the lower motor neurons [10,11]. Once inside inhibitory nerve terminals, tetanus toxin cleaves VAMP [11], thereby inhibiting the release of GABA and glycine. The result is a partial, functional denervation of the lower motor neurons, which leads to their hyperactivity and to increased muscle activity in the form of rigidity and spasms. It is not clear to what extent tetanus toxin in the spinal cord and brainstem is also taken up into excitatory nerve endings, such as those originating from the upper motor neurons, or those that convey impulses from the muscle spindles and constitute the sensory part of the simple, monosynaptic reflex arc of the tendon reflexes. Experiments in cats have shown tetanus toxin to augment central polysynaptic, but not monosynaptic, reflexes [12], suggesting a primary effect on inhibitory neurons. Studies *in vitro* and *in vivo* point to an early inhibition of inhibitory nerve endings and a later, or dose-dependent, involvement of excitatory nerve endings [13,14,15]. A temporary reduction in the number of GABAergic nerve terminals has been seen after injection of tetanus toxin into eye muscles of cats [16]; thus, the effect of tetanus toxin may be both biochemical and structural. 

## 3. Symptomatology of Tetanus

Tetanus toxin causes hyperactivity of voluntary muscles in the form of rigidity and spasms. Rigidity is the tonic, involuntary contraction of muscles, while spasms are shorter lasting muscle contractions that can be elicited by stretching of the muscles or by sensory stimulation; they are termed reflex spasms. For instance, rigidity of the temporal and masseter muscles leads to trismus (lockjaw), a highly reduced ability to open the mouth. Attempts at opening the mouth, e.g., during physical examination, may induce spasms that cause the complete clenching of the jaws.

Tetanus is categorized into generalized, neonatal (which is a generalized form in children less than one month), local, and cephalic (which is tetanus is localized to the head region). Generalized and neonatal tetanus affect muscles of the whole body and lead to opistotonus (the backward arching of the columna due to rigidity of the extensor muscles of the neck and back) and may cause respiratory failure and death due to rigidity and spasms of the laryngeal and respiratory muscles [1]. Local and cephalic tetanus account for only a minority of cases; however, they can develop into the generalized form.

Depending on whether it is local/cephalic or generalized/neonatal, tetanus typically manifests as trismus/lockjaw, risus sardonicus, dysphagia, neck stiffness, abdominal rigidity, and opistotonus, *i.e.*, hyperactivity of muscles of the head, neck, and trunk. The limbs tend to be less severely affected, but with full opistotonus there is also flexion of the arms and extension of the legs, as in a decorticate posture. Trismus is frequently the initial symptom in both local/cephalic and generalized tetanus [17,18], but the disease may present in any of the above-mentioned ways. In addition, general muscle ache, focal flaccid paralysis, and an array of unusual symptoms reflecting unusual patterns of neuronal inactivation, including diplopia [19], nystagmus [20], and vertigo [21], may occur.

The action of tetanus toxin is not confined to the motor system. Autonomic dysfunction with episodes of tachycardia, hypertension, and sweating, sometimes rapidly alternating with bradycardia and hypotension are common, especially in generalized tetanus [18,22,23]. Such symptoms are paralleled by dramatic increases in circulating adrenaline and noradrenaline [22,24], which may cause myocardial necrosis [25]. Autonomic symptoms tend to occur a week after the occurrence of motor symptoms. They have been interpreted to reflect an effect of tetanus toxin on the brainstem [24], although entry of tetanus toxin into preganglionic nerve terminals of the sympathetic nervous system has been demonstrated in experimental animals [26]; an effect of tetanus toxin on these neurons would be expected to cause autonomic dysregulation. With the advent of modern intensive care, which has made tetanus-mediated respiratory insufficiency a treatable condition, autonomic dysfunction has become a major cause of death in tetanus victims [2]. 

Sensory nerves may also become invaded by tetanus toxin [4,26], causing altered sensation, such as pain and allodynia [9,27]. It is unclear where this effect takes place, since experimental evidence suggests that the toxin is unable to pass spinal sensory ganglia [3]. Therefore, a sensory effect of the toxin should be peripheral. However, the vesicular release of neurotransmitters from sensory neurons occurs centrally, in the spinal cord or brainstem [28]. This apparent paradox may reflect the fact that altered sensation in tetanus is predominantly seen in the region of the head [9,27], *i.e.*, in the area of the (cranial) trigeminal nerve, the ganglion of which may differ from those of spinal sensory nerves with respect to axonal transport of tetanus toxin.

It is not known whether tetanus toxin that arrives in the brainstem spreads to structures involved in higher functions, such as cognition and mood regulation. Such symptoms are rarely reported. In a recent survey of 68 patients from Ethiopia, altered mentation was noted at an early stage in three patients, but it was not stated whether such symptoms could be attributed to the tetanus itself [18]. Sequelae of tetanus in the newborn include intellectual disability [29], which may suggest an effect of tetanus toxin on higher cerebral functions. Animal studies show clear effects of tetanus toxin on neuronal activity after focal application to the cerebral cortex [30], implying that if the toxin reaches the brain in tetanus victims, higher cerebral functions may become affected.

## 4. Treatment of Tetanus

Acute treatment of tetanus is based on wound cleaning and antibiotic eradication of Clostridium tetani, e.g., with intravenous metronidazole, 500 mg three times daily, or penicillin, 100,000–200,000 IU/kg/day [31,32]. Treatment is continued for seven to ten days. The notion that one should avoid penicillin because of a possible inhibition of the GABAA receptor, which could increase muscle rigidity, does not seem to be supported by studies [31]. Tetanus antitoxin is given once intramuscularly; doses of 500 IU, 3000 IU, or higher have been used, but it is debatable whether the higher doses are more effective [33]. The antitoxin is given to inactivate any free tetanus toxin. The toxin that has been taken up into nerve terminals is probably not available to the antitoxin. Therefore, muscle symptoms may develop further, although the clostridia have been eradicated and antitoxin has been given, because tetanus toxin continues to be transported axonally and trans-synaptically and to cleave VAMP. Intrathecal administration of antitoxin, e.g. via lumbar puncture, could inactivate tetanus toxin during its trans-synaptic transport; a meta-analysis indicated that intrathecal administration was superior to the intramuscular route with respect to survival [34]. Because immunity may not develop after an episode of tetanus, vaccination is included in the treatment.

Treatment of the muscular rigidity and spasms in tetanus is of vital importance, since this feature of the disease often interferes with respiration and is a likely cause of death [1,18]. Rigidity and spasms also cause severe pain, which stimulates muscle activity. Muscle relaxation is customarily achieved with benzodiazepines [35], which augment the effect of GABA on the GABAA receptors of lower motor neurons. Baclofen, which acts on GABAB receptors, may also be effective; when given intrathecally its sedative effect is avoided [36]. In the setting of an intensive care unit, propofol, another GABAA receptor modulator, may be used [37], as may non-depolarizing muscle relaxants (pancuronium, pipecuronium) [38], which act directly on the muscle motor end plates by competing for the acetylcholine binding site. Magnesium, a calcium antagonist that acts both by reducing acetylcholine release and by reducing the muscle response to acetylcholine [39,40,41], may be effective in relieving rigidity and spasms [42]. Magnesium also seems to reduce autonomic dysfunction [42,43], which is of importance, because anti-adrenergic drugs, especially beta-blockers, may produce untoward effects, including cardiac arrest [24]. Dantrolene, which binds to the ryanodine receptor in muscle and reduces calcium mobilization and thereby muscle contraction, is also in use [44,45].

Tetanus patients should be in a calm environment to avoid the triggering of spasms by noise or other sensory stimulation. This objective must be balanced against the need to avoid sensory deprivation, which predisposes to delirium, a condition that tetanus patients are prone to, given their often lengthy stays in intensive care units with mechanical ventilation and treatment with neuroactive drugs such as benzodiazepines and propofol [46].

Prophylaxis against tetanus consists of immunization with formaldehyde-inactivated tetanus toxin (toxoid) and measures to achieve good hygiene. For instance contamination of the umbilical stump of the newborn is a primary cause of neonatal tetanus. These issues are interrelated: a good immunization status in pregnant women leads to reduction in the prevalence of neonatal tetanus [47], because maternal anti-tetanus toxin antibodies are transferred across the placenta to the child in utero [48].

**Table 1:** Summary of case reports on the use of botulinum toxin against tetanus-induced muscle rigidity and spasms.

Reference	Age/sex	Cause/incubation time	Symptoms/Ablett grade	Botulinum toxin: treatment start ^a^/dose and injection sites	Time to onset/time to maximal effect
[49]	33/male	Nose wound/8 days	Trismus, dysphagia, ptosis. Cephalic tetanus/Ablett grade 3	15 days: Botox^®^ 50 IU in each masseter. Two injection sites per muscle.	3–4 days/2 weeks
[50]	28/male	I.v. drug abuse/unknown incubation time	Trismus, progressing to generalized tetanus/Ablett grade 3	>3 weeks: Dysport^® ^into left biceps + brachioradialis + both gastrocnemius muscles, total dose 1000 IU.	1 day/1 day
[45]	64/female	Hand wound/unknown incubation time	Generalized tetanus, including diffuse rigidity and pain, trismus, risus sardonicus, dysphagia/Ablett grade 3	3 weeks: Botox^® ^30 IU into each cricopharyngeal muscle with EMG ^b^	2 days/1 week
[45]	68/female	Leg wound/3 days	Generalized tetanus, including rigidity, opistotonus, trismus, risus sardonicus, dysarthria, dysphagia/Ablett grade 3	3 weeks: Botox^® ^30 IU into each cricopharyngeal muscle with EMG ^b^	2 days/1 week
[51]	80/female	Unknown entry and incubation time	Throat pain, dysphonia, neck rigidity, trismus. Cephalic tetanus/ Ablett grade 3	8 weeks: Botox^® ^75 IU into each sternocleidomastoideus, 25 IU into right trapezius, 50 IU into each levator scapulae	“responded well”
[9]	82/female	Forehead wound/11 days	Bell’s paresis, facial pain, trismus, tongue spasms. Cephalic tetanus/Ablett grade 3	5 days: Botox^® ^25 IU into each masseter and 10 IU into each temporalis muscle	3 days/3 weeks

a: time (days or weeks) after admission to the hospital. b: injections into the cricopharyngeal muscles were done with electromyographic (EMG) guidance. I.v.: intravenous. Please observe that Dysport^®^ and Botox^®^ cannot be compared directly with respect to dosage [52].

## 5. The Use of Botulinum Toxin against Tetanus-Induced Rigidity and Spasms

Botulinum toxins enter nerve terminals of lower motor neurons [6,7]. The toxins are zinc metalloproteinases that attack synaptic vesicle proteins, but they do so differentially: botulinum toxin A cleaves synaptosomal-associated protein (SNAP-25), botulinum toxins B, D, F, and G cleave synaptobrevin (which is also attacked by tetanus toxin); botulinum toxin C cleaves SNAP-25 and syntaxin [7]. Compared to tetanus toxin, the botulinum toxins undergo less axonal and trans-synaptic transport, although some transport does seem to occur [53,54]. Therefore, the effects of botulinum toxins remain fairly confined to the nerve terminals of lower motor neurons, inhibiting release of acetylcholine and activation of voluntary muscles. For this reason they may have a role in reducing the muscular hyperactivity in tetanus patients.

In six reported cases of tetanus, all with symptom severity that amounted to grade 3 in the Ablett symptom severity grading system, botulinum toxin A was used successfully to control muscle rigidity and spasms [9,45,49,50,51]. In three cases the tetanus was cephalic or fairly local; in three it was generalized (Table 1). In all cases beneficial effects of treatment were seen. However, only in one patient was the treatment given within the first week after admission to the hospital; in the remainder, botulinum toxin was given two to eight weeks after admission, although symptom severity was greatest in the earlier phase of the disease. Therefore, one cannot rule out the possibility that the improvement seen after treatment with botulinum toxin to some extent reflected the natural history of tetanus, including spontaneous resolution of muscle rigidity. In some cases [45,50,51] botulinum toxin was used to treat residual muscle rigidity that proved especially resistant to other muscle-relaxing therapies. 

In the four reports that give details on onset of action, improvement of rigidity was noted within one to four days. Maximal effect was reached after one to three weeks, except in one case, in which maximal effect was seen one day after injection of botulinum toxin (Table 1). The activity of botulinum toxin is reported to be increased by neuronal activity [55,56]. Theoretically, the action of botulinum toxin could be more rapid in tetanus, in which the activity of the lower motor neurons is much increased.

Dosage varied somewhat (Table 1), but resembled those commonly used to treat dystonia [57]. It should be noted that the two preparations of botulinum toxin A that were used, Botox^®^ and Dysport^®^ , are not equipotent and somewhat difficult to compare [52]. 

Only in one patient was treatment repeated (two months after the initial injection) [51]. In the remaining cases, the effect of botulinum toxin apparently outlasted the symptoms of tetanus. Only in one case was a side effect noted: a certain atrophy of the masseter muscles after botulinum toxin injection for trismus [49]. 

## 6. Advantages and Disadvantages of Botulinum Toxin Treatment in Tetanus

Trismus and dysphagia are early and common symptoms of tetanus, both generalized and cephalic. They constitute major hazards for the patient, irrespective of the threats of respiratory failure and autonomic dysfunction described above. Normal salivation predisposes to aspiration in a patient who cannot swallow normally or evacuate the mouth, wherefore aspiration and pneumonia commonly occur in tetanus [58,59]. Trismus further interferes with eating and with oral hygiene, which is an important issue, because the condition may last for many weeks, endangering dental health. Lastly, trismus is associated with involuntary tongue biting, which may be very painful [9]. 

The use of botulinum toxin to ameliorate tetanus-induced trismus must be considered a safe procedure, given that the masseter and temporalis muscles are at some distance from the larynx; injection into the cricopharyngeal muscles to alleviate dysphagia, in contrast, requires electromyographic guidance [45]. Treatment of trismus and dysphagia with botulinum toxin should probably be considered at an early stage in tetanus, because it may contribute to a more favorable course of the disease, reducing the risk of aspiration and pneumonia, allowing dental care, and, possibly, food intake. 

Injections into the trapezius, splenius capitis, levator scapulae and sternocleidomastoid muscles may alleviate painful neck rigidity; care must be taken to avoid neighboring vital structures, such as the carotid artery, and spread of botulinum toxin to the larynx. 

No information exists on the use of botulinum toxin on large truncal muscles in tetanus, such as the abdominal and erector spinae muscles, which are affected in generalized tetanus. Successful use of botulinum toxin to treat hyperactivity of abdominal muscles has been reported in Parkinson’s disease [60] and dystonia [61]. Botulinum toxin has been used for back pain syndromes with injection of the toxin into several levels of the erector spinae muscles in the L1–L5 area [62]. The total dose of botulinum toxin A (Botox^®^) in these cases was 240–500 IU. From such reports it seems feasible to use botulinum toxin in large truncal muscles affected by tetanus, although it must be emphasized that no clinical experience with such treatment has been published.

Additional advantages of botulinum toxin in the treatment of tetanus include the reduced need for muscle relaxants that affect consciousness [63] and the long lasting effect of botulinum toxins (>3 months) [64], which in most cases outlasts that of tetanus toxin.

Disadvantages of the botulinum toxin approach to tetanus include the difficulty of treating all affected muscle groups in generalized tetanus. Even so, botulinum toxin should be considered in generalized tetanus, in which the rigidity of certain muscle groups may pose a special therapeutic challenge. The slow onset of action and gradual increase in effect over one to three weeks necessitates simultaneous treatment with other muscle-relaxing drugs. The possibility of overdosing, the evidence of which may become manifest days after injection of botulinum toxin, makes it important to monitor patients closely. The protracted effect of botulinum toxin [64] implies that such side effects may be of some duration. A main obstacle to the use of botulinum toxin for tetanus may prove to be the cost of treatment, especially in generalized tetanus, in which large doses may be needed to reduce rigidity and spasms of large muscles.

## 7. Conclusions

There is limited experience with the use of botulinum toxin for the treatment of muscle rigidity and spasms in tetanus. However, from a few published case reports it would seem that such treatment is useful. This may be especially true for trismus, which constitutes a major problem in itself, predisposing to pulmonary aspiration, painful, involuntary tongue biting, anorexia, and dental caries. The treatment of trismus with botulinum toxin is probably a fairly safe procedure, since injection into the masseter and temporalis muscles can be achieved without endangering neighboring vital structures. However, the possibility of complications caused by distant spread of the toxin must be kept in mind. There is a general lack of randomized clinical trials with respect to both antibiotic [31,32] and muscle-relaxing therapies [35] in tetanus. It is to be hoped that the potential usefulness of botulinum toxin in the treatment of tetanus will lead to its evaluation in clinical trials.
